# Biopolymers form a gelatinous microlayer at the air-sea interface when Arctic sea ice melts

**DOI:** 10.1038/srep29465

**Published:** 2016-07-20

**Authors:** Luisa Galgani, Judith Piontek, Anja Engel

**Affiliations:** 1 GEOMAR Helmholtz Centre for Ocean Research Kiel, Düsternbrooker Weg 20, 24105 Kiel, Germany; 2 Alfred Wegener Institute Helmholtz Centre for Polar and Marine Research, Am Handelshafen 12, 27570 Bremerhaven, Germany

## Abstract

The interface layer between ocean and atmosphere is only a couple of micrometers thick but plays a critical role in climate relevant processes, including the air-sea exchange of gas and heat and the emission of primary organic aerosols (POA). Recent findings suggest that low-level cloud formation above the Arctic Ocean may be linked to organic polymers produced by marine microorganisms. Sea ice harbors high amounts of polymeric substances that are produced by cells growing within the sea-ice brine. Here, we report from a research cruise to the central Arctic Ocean in 2012. Our study shows that microbial polymers accumulate at the air-sea interface when the sea ice melts. Proteinaceous compounds represented the major fraction of polymers supporting the formation of a gelatinous interface microlayer and providing a hitherto unrecognized potential source of marine POA. Our study indicates a novel link between sea ice-ocean and atmosphere that may be sensitive to climate change.

The Arctic Ocean is one of the marine environments most sensitive to global warming^1^. In summer 2012, sea ice decreased to a historical minimum of 3.41 million square kilometers since satellite records from 1979 (National Snow and Ice Data Center, Colorado, USA).

Ice melting starts with the formation of visible pools, referred to as melt ponds, collecting melt water. As melting proceeds in the summer season, melt ponds penetrate the ice floe and represent an extending new habitat for sea ice microorganisms^2^. The progressively higher temperatures of recent decades have contributed not only to the decrease of ice coverage, but also to a reduction of ice thickness, thus accelerating melting and the loss of perennial ice. Nowadays, most of the Arctic ice is thin first year ice (FYI). FYI forms at the beginning of fall to melt in late spring or beginning of summer, and has progressively replaced multiyear ice (MYI) in extended areas of the Arctic Ocean^3^. Melt ponds lower the sea ice albedo, that is, the capacity of sea ice to reflect solar radiation. As a consequence, sea ice melting is accelerated, more heat is absorbed and stored in the ocean^4^ and the amount of light available for under-ice primary production increases^5^,^6^.

Besides warm oceanic currents, freezing or melting of sea ice is also regulated by surface energy fluxes influenced by low-level Arctic clouds^7^. Clouds form by vapor condensing around small aerosol particles called cloud condensation nuclei (CCN). Therefore, radiative forcing and earth’s albedo are determined by CCN concentration and density^8^, which, in turn, influence melting or freezing of Arctic ice. Clouds’ properties are of enormous importance in the Arctic environment, where sea ice is being rapidly lost leaving space to extended areas of open water. It has been suggested that a major source of Arctic CCN during the summer relies on nano- and micro gels of polysaccharides and other organic compounds exuded by surface water phytoplankton^9^. Marine primary organic aerosols (POA) can account for ~63% of total submicron aerosol mass during phytoplankton bloom periods, ~45% of which is represented by water insoluble colloids^10^. Especially towards the end of the bloom, a large fraction of organic matter is released to seawater when cells lyse due to viral infection, a process potentially representing a biological control of POA formation at the nanoscale^11^. Besides bloom-related process, the large background reservoir of organic carbon in surface waters may be a more steady marine source for POA^12^. The emission of sea spray from the immediate interface layer between the ocean and atmosphere, referred to as sea surface microlayer (SML), is one initial step to POA formation^13^. The SML is a 20–100 μm thin, gelatinous film^14^, enriched in exopolymeric substances that are believed to be an important source for atmospheric aerosols^9^,^15^ and are potentially capable for nucleating ice in clouds^16^. At times of high biological activity, water insoluble^10^ and water soluble organic components in POA^17^ have been shown to resemble organic components observed in the SML^17^,^18^.

Marine nano- and micro gels form from dissolved exopolymers of polysaccharidic or proteinaceous composition, released by marine microorganisms during exudation, degradation and lytic processes^19^,^20^. Marine gels cover the size range from nm to several μm^21^, with gels (>0.4 μm) being defined as transparent exopolymer particles (TEP, polysaccharidic) and as Coomassie stainable particles (CSP, proteinaceous)^22^,^23^,^24^. Both ice algae and bacteria produce copious amounts of exopolymers as a survival strategy to control their adherence in the brine channels and to buffer their immediate environment against physical and physico-chemical stress during freezing and melting processes, high salinity and low temperatures^25^,^26^,^27^.

Sea ice is a large reservoir of organic substances: through the release of exopolymers and antifreeze proteins, sympagic algae and bacteria directly influence the properties of the brine channels increasing the abundance of pores and changing the structure of the ice^25^,^28^. During bloom periods, exopolymers include high concentrations of dissolved uronic acids (DURA) and neutral sugars^27^,^29^. Outside the algal growing season, bacteria are an important source of exopolymers^25^,^27^. Besides the autochthonous microbial production of biomass, dissolved and particulate organic matter (DOM, POM) already present in the seawater become incorporated during ice formation and selectively released during the melt season^30^,^31^.

FYI holds most biomass near the water-air interface^30^. Because of a thinner sea ice cover and a higher light transmittance, widespread under-ice phytoplankton blooms have been observed^32^,^33^, with an intensified release of DOM, including gel precursors, from phytoplankton cells in surface waters^34^. Polysaccharidic gels in the SML potentially act as source for airborne particles^9^,^18^, but so far it is unknown to which extent sea ice exopolymers may contibute to the organic fraction of the SML. The production of sea ice exopolymers is not always coupled to the algal growing season^25^,^27^, and the composition of POA does not reflect highest chlorophyll *a* concentrations and algal blooms^12^. Therefore, there might be an additional source of organic matter accumulating in the SML that may alter SML structure and contribute to the organic fraction of POA in the high Arctic.

In this study, we aimed at understanding how the retreat of Arctic sea ice affects the organic composition of the SML, with implications for POA emission. During a cruise with the RV POLARSTERN (ARK XXVII/3) to the central Arctic Ocean, we sampled the SML from different sites: shallow and open melt ponds and open sea across 7 different ice stations (Fig. 1). The cruise took place in the eastern-central ice covered basins between 82° to 89°N and 30° to 130°E between 2^nd^ of August and 8^th^ of October, 2012. The study area was characterized by a domination of FYI (>95%) and melt pond coverage of approximately 30–40%^33^.

## Results

### Surface microlayer composition from melt ponds to open sea

**Figure 1:**
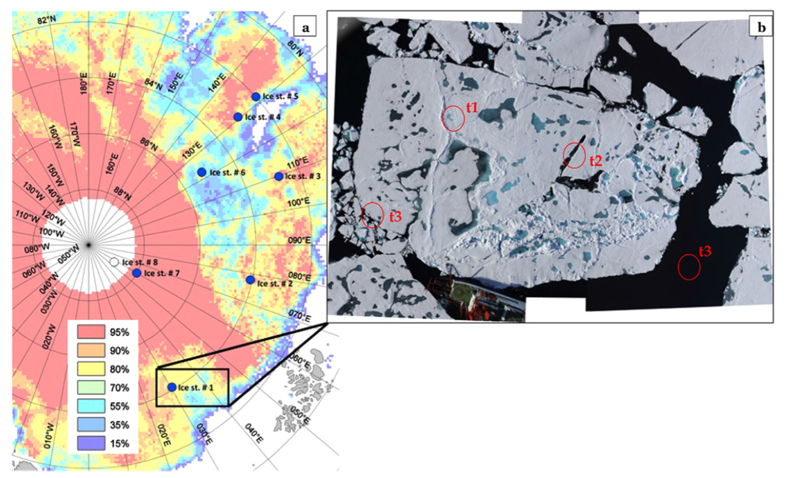
(**a**) Ice stations of RV *Polarstern* during cruise leg ARK 27-3 (IceArc) and ice cover in August 2012 (%). Map courtesy of S. Albrecht, Fielax, modified after Boetius *et al*.^33^. Reprinted with permission from AAAS. **(b)** The three different types of sampling sites are exemplified for Ice Station #1 with t1: freshwater melt ponds, t2: open melt ponds and t3: open sea. t1 and t2 are actual sampled melt ponds, while t3 presents two examples for open sea type, sampled at the ice edge or with a zodiac. Image courtesy of S. Hendricks, Sea-Ice Physics, AWI.

Based on differences in morphology and physical parameters such as depth, salinity and formation time, we have identified three categories of sampling sites: t1) freshwater melt ponds, t2) open melt ponds and t3) open sea samples (Fig. 1). Sea ice melting starts with the accumulation of freshwater from melting snow, and shallow ponds are created (e.g., t1). As melting proceeds, shallow ponds become deeper holes in the sea ice where the initial freshwater from melted snow and surface ice layers mixes with more saline water from the bottom ice and a vertical salinity gradient in the ponds is established (e.g., t2). As the pack ice breaks and completely melts, we assist to the appearance of open leads and open water (e.g., t3).

**Figure 2:**
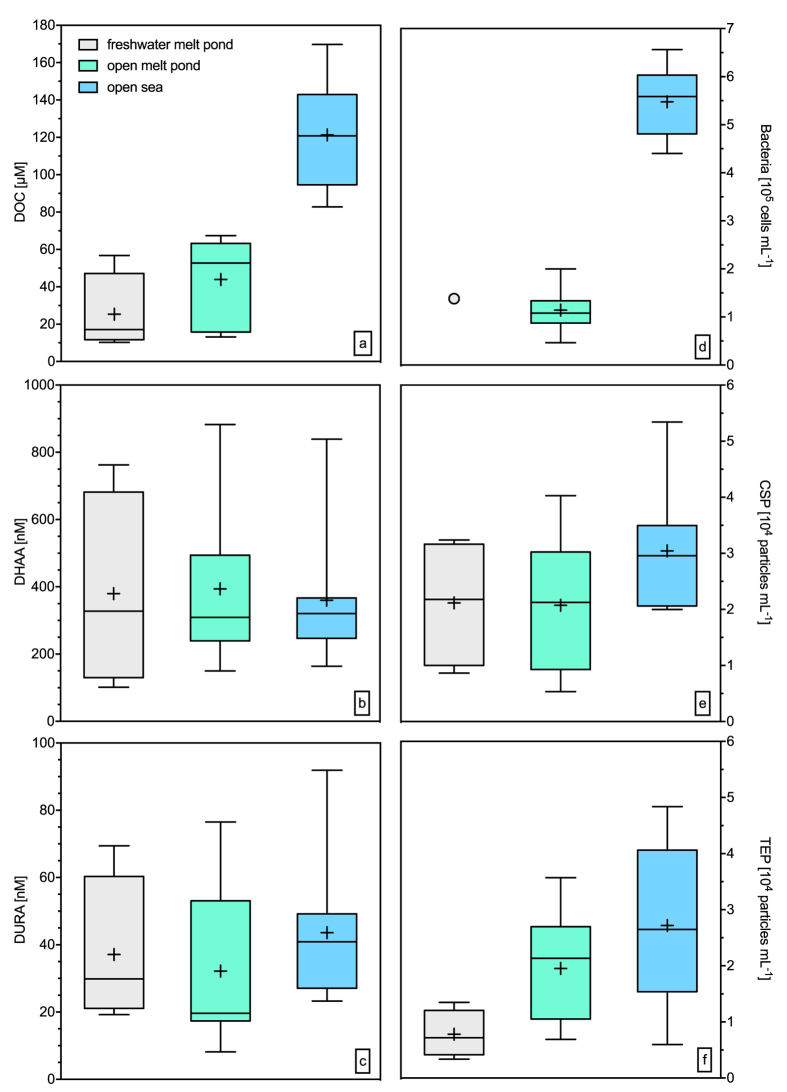
Box plots for biogenic compounds of the SML: (**a**) Dissolved organic carbon (DOC), (**b**) Dissolved hydrolysable amino acids (DHAA), (**c**) dissolved uronic acids (DURA), (**d**) bacterial abundance (just one sample), (**e**) Coomassie stainable particles (CSP), (**f**) transparent exopolymer particles (TEP). The horizontal lines of the boxes represent 25%, 50% (median) and 75% percentiles (from bottom to top). In the boxes, crosses represent the mean. Whiskers represent minimum and maximum values.

**Figure 3:**
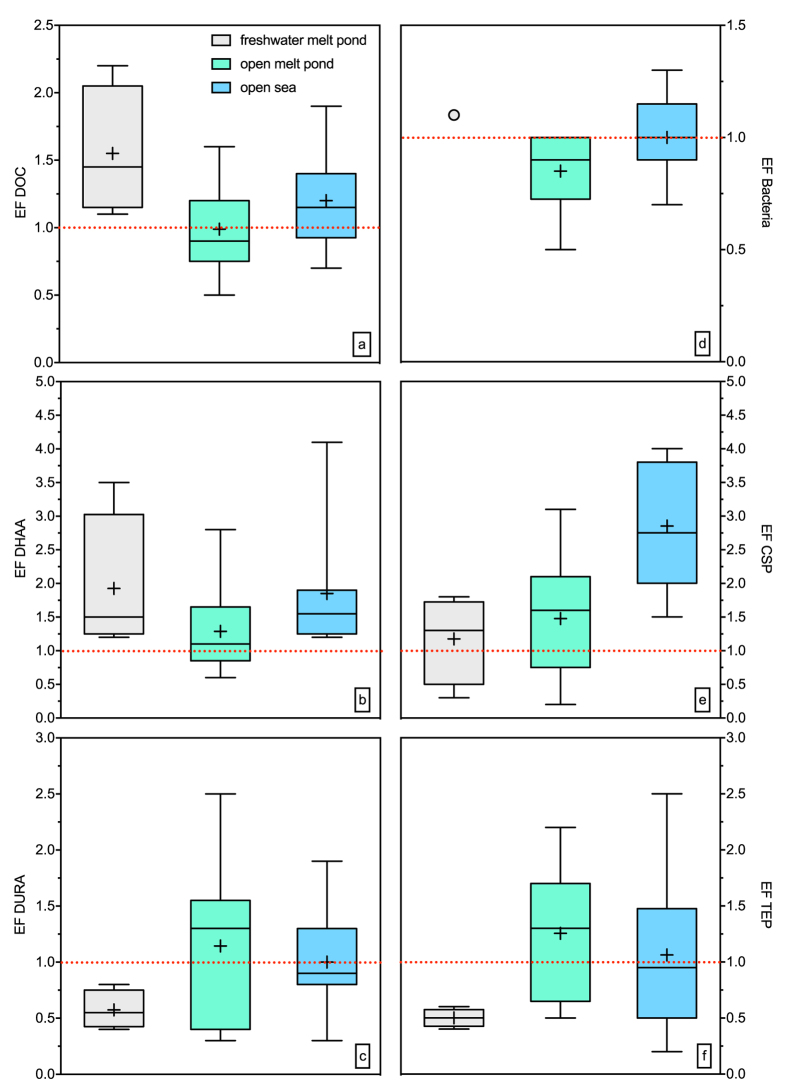
Enrichment factors (EF) for (**a**) dissolved organic carbon (DOC), (**b**) EF for dissolved hydrolysable amino acids (DHAA), (**c**) EF for dissolved uronic acids (DURA), (**d**) EF for bacterial abundance, (**e**) EF for Coomassie stainable particles (CSP), (**f**) EF for transparent exopolymer particles (TEP). The horizontal lines of the boxes represent 25%, 50% (median) and 75% percentiles (from bottom to top). In the boxes, crosses represent the mean. Whiskers represent minimum and maximum values.

The concentration of dissolved organic carbon (DOC) increased in the transition from freshwater melt ponds to the open sea (Fig. 2a) as confirmed by significant differences between the sampling types (Holm-Sidak one-way ANOVA, *p* < 0.01; Table S3). DOC concentrations in the SML and underlying water (ULW) were not significantly different (*MWRS, p* > 0.05, *n* = 21). DOC concentrations were significantly related to salinity both in the SML (*C* = 0.80, *n* = 14, *p* < 0.01) and ULW (*C* = 0.70, *n* = 14, *p* < 0.01). Enrichment factors (EF) for DOC in the SML differed between the sample types (Fig. 3a, Table S1), indicating slight enrichment in SML of t1 and t3, whilst no consistent enrichment was found in t2. The average concentrations of dissolved hydrolysable amino acids (DHAA) were only slightly different across sampling sites but showed high variability in t1 (Fig. 2b, Table S3). At most stations, DHAA were enriched in the SML (Fig. 3b, Table S1), yielding significant differences between concentrations in the SML and ULW (*MWRS, p* = 0.021, *n* = 21).

Dissolved uronic acids (DURA) concentrations ranged from 8.2 nM to 92 nM in the SML and from 6.6 nM to 121 nM in the ULW, with no significant differences. These concentrations represent the lower end of previously reported data from Arctic ice cores^27^ (Fig. 2c, Table S3). Overall, DURA concentration was positively correlated with TEP abundance (*C* = 0.52, *n* = 21, *p* = 0.02), emphasizing DURA as an important source for TEP formation. However, except for a few sites, DURA were generally depleted in the SML of our study (Fig. 3c, Table S1).

Bacterial abundance in the SML of freshwater and open melt ponds was low compared to open sea samples (Fig. 2d). In general, bacteria were not enriched in the SML (Fig. 3d, Table S1). Bacteria were significantly related to DOC (*C* = 0.73, *n* = 17, *p* < 0.01), TEP abundance (*C* = 0.52, *n* = 17, *p* = 0.03) and TEP size (*C* = 0.75, *n* = 17, *p* < 0.01), indicating a general control by substrate availability. Overall, enrichment factors for DOC, DHAA, carbohydrates, bacteria and TEP observed during this study agree well with earlier findings^14^,^15^,^18^.

### Proteinaceous compounds dominate SML gels

Arctic surface microlayers were characterized by a high abundance of proteinaceous gels, i.e. CSP (Fig. 2e), and, as described, most sampling sites showed CSP enrichment in the SML (Fig. 3e, Table S1). CSP in the SML ranged from 5 × 10^3^ mL^−1^ to 53 × 10^3^ mL^−1^ (Table S2), significantly higher than in ULW (*MWRS, n* = 21, *p* = 0.016). Higher CSP abundances in the SML with respect to ULW were observed also in the submicron size range (0.4–1 μm) (*MWRS, n* = 21, *p* = 0.013).

TEP abundance ranged from 3 × 10^3^ mL^−1^ to 48 × 10^3^ mL^−1^ (Table S2), with no significant differences between SML and ULW (Fig. 3f, Table S1). TEP abundance, volume concentration and total area in the SML increased in the transition from freshwater melt ponds to open sea samples (one-way ANOVA, *p* = 0.035, *p* = 0.01 and *p* = 0.007 for abundance, volume concentration and area respectively), along with increasing salinity.

Thus, CSP were more abundant in the SML than TEP. They also comprised a significantly larger total average area in the SML (110 ± 72 mm^2^ L^−1^) than TEP (53 ± 52 mm^2^ L^−1^) (Fig. S1, *MWRS, n* = 21, *p* = 0.001). Moreover, the submicron fraction contained significantly more CSP than TEP (*MWRS, n* = 21, *p* = 0.003, Fig. S1).

In general, highest gel abundance was observed in open sea SML, on average 30 ± 11 × 10^3^  mL^−1^ for CSP and 27 ± 14 × 10^3^  mL^−1^ for TEP. However, the most pronounced difference between CSP and TEP abundance was observed in freshwater melt ponds (Fig. 2, Table S2), with average values of 21 ± 11.2 × 10^3^  mL^−1^ CSP and 7.8 ± 4.1 × 10^3^  mL^−1^ TEP. At these sites, on average 74 ± 8.5% of TEP and 69 ± 5.5% of CSP were in the submicron size range (Table S2). So far, only a few studies investigated proteinaceous gels in the SML^35^,^36^,^37^, but found no significant differences between CSP and TEP. In contrast, selective enrichment of CSP observed in the SML of melt ponds suggests a specific protein-rich source in the ice.

Size-frequency distributions of gels may be described by the spectral slope (δ) of the distribution curve. A less negative δ implies that there is a higher fraction of gels in larger size classes. In the SML, average δ for TEP (−3.18 ± 0.2) and CSP (−2.91 ± 0.12) were significantly different (Fig. S2, *MWRS, n* = 21, *p* < 0.001), indicating a higher proportion of larger particles in CSP. The 50% percentile of all CSP fell in the range 0.5–1.5 μm (SML) and 0.3–1.1 μm (ULW). TEP were smaller, mostly in the submicron fraction; the 50% percentile ranged from 0.5 μm to 0.8 μm (SML) and 0.3 μm to 0.6 μm (ULW).

Both in the SML and ULW the average TEP size increased with increasing salinity (*C* = 0.70, *n* = 14, *p* < 0.01, and *C* = 0.68, *n* = 14, *p* < 0.01, respectively), supporting the idea that TEP formation is enhanced when divalent cations such as Ca^2+^ become increasingly abundant at higher salinity^38^.

No apparent correlation with salinity was observed for CSP size. However, CSP dominated the submicron gel fraction in the SML of the Arctic Ocean, which is relevant for POA emission. Moreover, CSP were ubiquitously present, independently of the sampling site, and even at lower DOC concentrations.

### Melt ponds as a source of organic exopolymers

**Figure 4:**
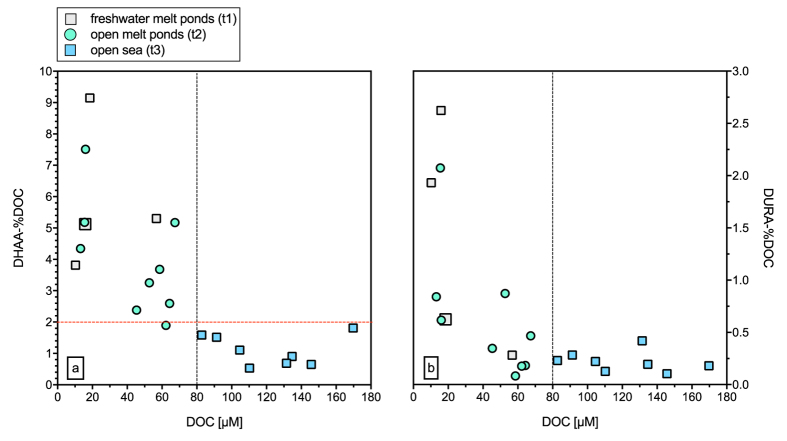
(**a**) Carbon-normalized yields of dissolved hydrolysable amino acids (DHAA-%DOC) and **(b)** carbon-normalized yields of dissolved uronic acids (DURA-%DOC) against DOC concentrations in the SML. The horizontal dashed red line in **(a)** indicates DHAA-%DOC of 2% as threshold between labile and refractory DOM according to Davis and Benner, (2007). The vertical dashed black line at 80 μM DOC **(a,b)** indicates the average refractory DOC background in surface waters (<100 m depth) of the Central Arctic Ocean (above 80°N)^73^. Both DHAA-%DOC and DURA-%DOC were inversely related to DOC concentration, with r^2^ = 0.66 and *p* < 0.01 (DHAA), and r^2^ = 0.60 and *p* < 0.01(DURA), both as 3^rd^ order polynomial regressions.

The yield of DHAA in DOC (DHAA-%DOC) may serve as an indicator of the diagentic state of DOM. A value of ~2% DHAA in DOC has been suggested as threshold separating refractory from labile and semi-labile organic matter^39^. Accordingly, DOM sampled in the SML of melt ponds fell into the semi-labile and labile category, while the SML of open sea sites, whether at the ice edge or in the open ocean, contained more refractory DOM (Fig. 4, Table S3). Higher percentages of labile DOM in melt ponds compared to more saline water can be partly explained by the absence of a large, old DOM pool like in the open ocean, as snow and ice have rather low DOC concentrations of 16–58 μM^40^. On the other hand, melting releases microbial exopolymers that accumulate in the sea ice brine during winter^41^, as well as substances freshly produced by phytoplankton when light becomes available for photosynthesis during summer^42^. DHAA-%DOC yields were quite high in melt ponds compared to open sea, and similar to values found in Arctic sea ice melt water (Table S3)^43^, reflecting the short lifetime of melt ponds as new environments.

Including all SML data, an inverse relationship was observed between DOC concentration and DHAA-%DOC representing the transition from fresh melt ponds to open ocean (Fig. 4, with r^2^ = 0.66, *p* < 0.01). As for DHAA-%DOC, the DOC fraction of DURA also showed an inverse relationship with DOC concentrations in the SML (Fig. 4, with r^2^ = 0.60, *p* < 0.01). DURA-%DOC was highest in melt ponds, declining towards the open sea (Table S3).

## Discussion

The link between ocean biology and atmospheric processes is one of the most intriguing open questions in climate sciences. Entrainment of organic matter in sea-spray and the subsequent nucleation of clouds^16^ have been linked to phytoplankton productivity^10^, to oceanic organic carbon reservoirs^12^ and more recently, to nanoscale biological processes, such as viral infection, driving the demise of phytoplankton blooms^11^. Arctic sea ice interposes between ocean biology and the atmosphere and its melting releases organic compounds that may accumulate in the SML. Therefore, sea ice decline potentially influences the organic content of sea-spray aerosols in the region.

Among the organic compounds observed, the enrichment of amino acids in the SML has been previously described for different marine environments^14^. DHAA are labile to semi-labile substrates taken up by bacteria within days to weeks^44^. Thus, DHAA accumulation in the SML may point to a reduced bacterial activity and related to high light intensities and UV stress^45^, specifically in melt ponds. While DHAA enrichment in the SML has been reported earlier, gels with a prevalent proteinaceous composition, like CSP, have only been determined in few studies in temperate and tropical seas^35^,^36^,^37^. This is the first study showing high abundances of proteinaceous gels in the central Arctic Ocean. CSP were found in higher amount and volume fraction with respect to TEP, even in melt ponds of recent formation and at lower DOC concentrations.

Here, both polysaccharidic and proteinaceous marine gels contributed substantially to submicron particles in the SML, but CSP dominated the overall abundance, implying a relevant protein-like gelatinous source for POA. Recent observations point to a high contribution of proteinaceous compounds to POA during spring and summer in the high Arctic^46^, with high concentrations of free amino acids in the aerosol ultrafine fraction (<0.49 μm) with a possible biological origin in the marine surface layer^47^. In the central Arctic Ocean, at low wind speeds and in the absence of breaking waves, the transfer of organic compounds from the SML to the atmosphere may be mediated by bubble rising and bursting^48^. Rising of bubbles originates from density gradients between a colder thin surface layer and the warmer underlying water, initiating a buoyancy-driven mixing, as well as from respiration of marine organisms and the release of trapped bubbles from within the melting ice^48^.

Where do the organics enriching the Arctic SML come from? One likely source is the sea ice itself. To increase the habitability of sea ice, microorganisms such as the diatom *Melosira arctica* release extracellular polymeric substances (EPS) to the ice brine and, thereby, modify sea ice properties such as porosity, salt retention, and sea ice structure^25^,^27^,^28^,^41^. EPS include ice-binding proteins that can also be found in ice-adapted bacteria^49^. As sea ice melts, these organic substances in the brine are released with the melt water and may accumulate in the SML.

The enrichment of proteinaceous compounds in the SML may also be related to microbial lysis through osmotic shock, or to the direct exposure to UV radiation as soon as the ice melts^50^,^51^. UV is able to promote the photochemical breakdown of particulate and refractory organic matter (photodissolution)^52^,^53^, thus increasing the concentration of biologically available DOM. In this perspective, the combined effects of microbial exudates and photodissolution may provide a new source of fresh DOM into melt ponds during summer, as suggested by the high yield of DHAA-%DOC and DURA-%DOC in our SML samples.

DURA are known as important component of TEP that are considered as mucopolysaccharide gels, whose structure is formed due to divalent cation (Ca^2+^, Mg^2+^) bridging between uronic acids, as well as by half-ester sulfate bridging between individual molecules^21^,^22^,^54^. It is assumed then that DURA in seawater represent precursor material for TEP formation^21^,^22^. A lower TEP abundance in freshwater melt ponds may be related to the lower concentration of cations, in accordance with the observed increasing amount of TEP with increasing salinity^38^. In seawater, the accumulation of DURA can be related to the relatively long turn-over time of high-molecular weight heteropolysaccharides (months-years) that are considered as semi-labile DOM^55^. In comparison, DURA concentration in melt ponds was relatively high given the young age of melt ponds (days-weeks) and suggested significant release from sea ice. Similarly, it is known that CSP contain amino acids, as they are stained by the amino-acid specific dye Coomassie Brilliant Blue^23^. Thus, DHAA likely contain precursor material for CSP formation. However, the mechanisms of proteinaceous gel particles formation may be more independent from cations, which could explain why we did not see a gradient in CSP abundance towards the open sea.

Compared to freshwater melt ponds, DHAA were found in similar concentration in open melt ponds (t2-type), which also showed higher DOC concentrations, higher bacterial abundance, and higher number of TEP. Open melt ponds are connected to the surrounding ocean, but are not disturbed by waves and usually have lower surface salinity^2^. In late summer, when temperatures drop fast below zero, open ponds refreeze incorporating algal aggregates of *M. arctica* and other phytoplankton species into the soft iced surfaces. We observed algal aggregates in soft surface ice of open melt ponds during our cruise^34^,^56^. The observed enrichment of polysaccharide components (DURA, TEP) in open melt ponds may therefore be directly related to primary productivity and algal biomass of *M. arctica*, as suggested by many studies^9^,^17^,^27^,^28^.

When temperature drops and melt ponds rapidly freeze up, sea ice brine is expelled upwards to the surface of FYI forming a highly saline skim, which feeds frost flowers, i.e. ice crystals with branched structures at the interface between a warmer surface and a colder atmosphere^57^. Additionally to the SML, frost flowers represent another ice/ocean-atmosphere interface. During melting they may support the organic fraction of POA as they host microbial communities and EPS found in the brine^58^. When melt ponds freeze up again, ikaite crystals (calcium carbonate) precipitate in frost flowers, a process that releases CO_2_ to the atmosphere^58^.

The air-sea exchange of CO_2_ and other gases is controlled by organics in the SML that physically and chemically alter gas exchange coefficients^59^. Additionally, SML bacteria may provide a net source of CO_2_ through respiration^60^. Melt pond SMLs thus represent dynamic exchange interfaces between the sea ice biology and the overlying atmosphere. Organic polymers in the SML of recently formed shallow melt ponds may be incorporated as gels into frost flowers and brine skim, potentially representing an important source for arctic POA during melting periods, and contributing to the exchange of CO_2_ during both melting and freeze up.

Resuming, during the Arctic summer, the melting of sea ice and the increasing light availability promote a release of organic exopolymers into the newly forming melt ponds, as well as primary production is boosted both in deeper melt ponds as well as in open waters. The summer biological production and release of organic matter from the melting ice may provide the exopolymers for the establishment of an organics-enriched SML. At the end of the summer, when the pack ice forms again, the organics in seawater may get incorporated into the ice and at the same time, microbial survival metabolism provides a new source of polymeric DOM in the ice brine, that will be released during the next melting season.

As we observed DHAA in similar concentrations in all stages of ice melting, and CSP dominating gel abundance, we suggest that the transition towards FYI in the central Arctic Ocean and the increasing number of melt ponds during the summer supports proteinaceous compounds in the SML and their contribution to Arctic aerosol mass.

Future changes in the Arctic Ocean affecting sea ice and ocean biology will likely influence organic matter concentration in the SML, with potential consequences for POA composition and air-sea gas exchange. Arctic change does not only account for faster sea ice melting and the loss of MYI, it also implies temperature and CO_2_ effects on primary production, DOM accumulation and turnover, in turn affecting exopolymer release and carbon flow^61^,^62^. Our findings suggests that a gel-like SML^63^, dominated by proteinaceous compounds and distinct from the ocean underneath, might be ubiquitously present in the Arctic, from newly formed melt ponds to open sea. We are just at the beginning to explore the biogeochemical interactions between sea ice, ocean and atmosphere in this region. Understanding processes in the SML as direct interface could make an important contribution to better represent and predict temporal and spatial variability in biogeochemical and climate models.

## Methods

### Sampling site and procedure

Samples were collected at 21 sites across seven different ice stations (Fig. 1). These sites were categorized in three different types: t1) freshwater melt ponds mostly of melted snow, and very shallow (~50 cm) depth (4 sites), t2) open, deeper melt ponds and partly connected with the ocean with an accentuated ice melting process (9 sites), t3) open sea samples as (8 sites). The SML was sampled from all different locations with a borosilicate glass plate of 250 × 500 × 4 mm dimensions with an effective sampling area of 2000 cm^2^ according to the original approach described by Harvey and Burzell^64^ and in line with recent guidelines for surface samples^65^. SML sample was wiped out with Teflon blades, which allowed the water to be collected into glass bottles previously pre-acid washed (HCl 10%) and thoroughly rinsed with Milli-Q water. Underlying water (ULW) samples were collected directly after the SML-sampling with pre-acid washed and MilliQ water rinsed glass bottles, from approximately 20~25 cm below the surface, closing the bottle underwater. Open water samples were collected from a zodiac between the ice floes at about 4 nautical miles from the ship in order to minimize contamination. During these operations, the outboard engine was turned off and samples were collected upwind to avoid any contamination with the zodiac itself. The reference thickness of the layer collected (*d*, μm) was on average 54.8 ± 4.8 μm, and was calculated according to


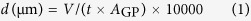


where *V* is the water volume collected (cm^3^), (*t*) the number of dips with the glass plate, and *A*_GP_ (cm^2^) the a total area of the glass plate, considering both sides.

Enrichment factors between SML and underlying water were calculated as follows:


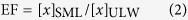


where *[x]* is the concentration of a given parameter in the SML or in the underlying water (*ULW*)^66^.

Enrichment factors are the ratios of two quantities, and, therefore, include the propagation of the individual standard deviations of these quantities. Standard deviations for replicate analyses for dissolved organic carbon (DOC), dissolved hydrolysable amino acids (DHAA), dissolved uronic acids (DURA) and bacterial abundance in SML and underlying water samples were derived from replicate analyses. The standard deviation of enrichment factors was calculated from standard deviations of the microlayer and underlying water samples according to the Gaussian law of error propagation, and the reference values (SD %) are given in Table S1. For TEP and CSP, we assumed a constant conservative SD of 50% since no replicate measurements were made.

### Dissolved organic carbon

Samples for dissolved organic carbon (DOC) were prepared by filtering seawater through pre-combusted (8 h, 500 °C) GF/F filters (0.7 μm, Whatman) and filling 20 mL into pre-combusted (8 h, 500 °C) glass ampoules successively acidified with 80 μL of 85% phosphoric acid (H_3_PO_4_). DOC samples were stored for three months at 0–2 °C until analysis. DOC concentrations were determined by high temperature catalytic oxidation modified after Sugimura and Suzuki^67^ with a Shimadzu TOC-VCSH analyzer and as the mean of quadruplicate measurements. Details on the analytical procedure are available in the supporting information.

### Dissolved amino acids

For dissolved hydrolysable amino acids (DHAA) analysis, samples were prepared by filtering seawater through 0.45 μm GHP membranes (Acrodisk, Pall Corporation) and sub-sampling 15 mL into pre-combusted (8 h, 500 °C) scintillation vials. The analysis was performed according to Dittmar and colleagues^68^ and Lindroth and Mopper^69^. Analytical details are given in the supporting information. The carbon content of DHAA was normalized to the amount of dissolved organic carbon and reported as DHAA-%DOC.

### Dissolved uronic acids

Samples for high molecular weight (>1 kDa) dissolved uronic acids (DURA) were prepared by filtering seawater through 0.45 μm GHP membranes (Acrodisk, Pall Corporation) and sub-sampling 15 mL into pre-combusted (8 h, 500 °C) scintillation vials. Samples were kept frozen at −20 °C until analysis. The analysis was conducted according to Engel and Händel^70^, and the analytical process is described in the supporting information. The carbon content of DURA was normalized to the amount of dissolved organic carbon and reported as DURA-%DOC.

### Bacterial abundance

For bacterial abundance, 4 mL of sample were fixed with 200 μL glutaraldehyde (25%) and stored at −20 °C until enumeration within six months from collection. Abundance was determined after staining with SYBR Green I (Invitrogen) and the analysis performed with a flow cytometer (FACSCalibur, Becton Dickinson). Bacterial cell numbers were estimated by manual gating of the bacterial subpopulation in the cytogram of side scatter versus green fluorescence, with a standard deviation <3% between replicate measurements. Yellow-green fluorescent latex beads (Polyscience) and TruCount beads (Becton Dickinson) were used to normalize the counted events to volume^71^.

### Marine gel particles: TEP and CSP

Marine gels were determined microscopically with the CytoClear slide technique after Engel^22^. Sampling TEP with a glass plate does not introduce biases in TEP concentration^72^, and we assumed this to be valid for CSP particles too. Twenty to 80 mL of sample were filtered onto polycarbonate filters (Nucleopore) of 0.4 μm pore size (Whatmann) in two replicates, and immediately stained with 1 ml of Alcian Blue (AB) solution for TEP or Coomassie Brilliant Blue G (CBBG) for CSP and mounted onto CytoClear slides. CytoClear slides were stored at −20 °C until microscopy. For each slide, thirty images were taken randomly per filter cross section at 200x magnification with a light microscope equipped with a digital AxioCam HRc camera (Zeiss). The analysis of the cross-sectional area of marine gels was performed with an image analysis software (ImageJ, US National Institutes of Health) allowing the calculation of the equivalent spherical diameter (ESD) of individual particles, particles number, volume and total area. Size-frequency distribution analysis and determination of TEP carbon content are described in details in the supporting information.

### Statistical analysis

Calculation of Pearson and Spearman Rank Order Correlation Coefficient (*C*), 3^rd^ order polynomial regression, and statistical test like Mann-Whitney Rank Sum (*MWRS*) test were performed with SigmaPlot (Systat Software Inc.) package and Prism7 (GraphPad Software Inc.). Statistical significance was accepted for *p* < 0.05.

## Additional Information

**How to cite this article**: Galgani, L. *et al*. Biopolymers form a gelatinous microlayer at the air-sea interface when Arctic sea ice melts. *Sci. Rep.* **6**, 29465; doi: 10.1038/srep29465 (2016).

## Supplementary Material

Supplementary Information
