# Supplementary material for: Biopolymers form a gelatinous microlayer at the air-sea interface when Arctic sea ice melts
Source: Sci Rep. 2016 Jul 20;6:29465. doi: 10.1038/srep29465 (PMC4951643; doi:10.1038/srep29465)
Supplement: Supplementary Information [file srep29465-s1.pdf]

Supplementary Information to the manuscript

“Biopolymers form a gelatinous microlayer at the air-sea interface when Arctic sea-ice melts”

Luisa Galgani[1,2,3], Judith Piontek[1] and Anja Engel[1,*]

[1]GEOMAR Helmholtz Centre for Ocean Research Kiel, Düsternbrooker Weg 20, 24105 Kiel, Germany

[2]Alfred Wegener Institute Helmholtz Centre for Polar and Marine Research, Am Handelshafen 12, 27570 Bremerhaven, Germany

[3]now at Università degli Studi di Siena, Department of Biotechnology, Chemistry and Pharmacy, Via A. Moro 2, 53100 Siena, Italy

*corresponding author:

Anja Engel

Düsternbrooker Weg 20

24105 Kiel, Germany

##49-431-6001510

aengel@geomar.de

Analytical procedures

Dissolved organic carbon

The calibration for DOC was made with the TOC standard solution (1000 mg L^{-1} from Merck-Certipur® ref. 109017) over the measurement range of 0 - 600 µM C, and Deep Sea Water reference (Batch 10 – 2010 Lot #05-10) from Hansell laboratory, University of Miami, Florida. Every measurement day, ultrapure (Milli-Q) water was used to determine the instrument blank, which was accepted for values < 1 µmol C L^{-1}. DOC concentration was determined in each sample from 5 to 8 injections. The precision was < 4% estimated as the standard deviation of replicate measurements divided by the mean.

Dissolved hydrolysable amino acids

For dissolved hydrolysable amino acids (DHAA), 1 mL of sample and 1 mL of 30% hydrochloric acid (HCl) (Merck, suprapure) were hydrolyzed in sealed ampoules at 100°C for 20h. The hydrolysate was dried in a microwave under nitrogen atmosphere and was washed twice with 0.5 mL of ultrapure water to remove the HCl. Finally the samples were re-dissolved in 1 mL ultrapure water. Amino acids were separated by HPLC (1260, Agilent) equipped with a C18 column (Phenomenex Kinetex, 2.6µm, 150 x 4.6mm) after in-line derivatization (2 min) with o-phthaldialdehyde and mercaptoethanol. For solvent A 0.1 M sodiumdihydrogenphosphate was adjusted to pH 7 with sodium hydroxide (NaOH) and premixed with acetonitrile (19:1 v/v). Solvent B was acetonitrile. A linear gradient was run starting from 6% solvent B to 27% solvent B in 40 min at a flow rate of 0.8 mL/min. The following standard amino acids were used: aspartic acid (AsX), glutamic acid (GlX), serine (Ser), arginine (Arg), glycine (Gly), threonine (Thr), alanine (Ala), tyrosine (Tyr), valine (Val), phenylalanine (Phe), isoleucine (Ileu), leucine (Leu), γ- amino butyric acid (GABA). α- amino butyric acid was used as an internal standard to account for losses during handling. Standards were run in the beginning as well as after each 5th sample. The detection limit for individual amino acids was 2 nmol monomer L^{-1}. The precision was <5%, estimated as the standard deviation of replicate measurements divided by the mean.

Dissolved uronic acids

Samples for dissolved uronic acids (DURA) were desalinated by membrane dialysis (1 kDa MWCO, Spectra Por) for 5 h at 6 °C, hydrolyzed for 20 h at 100°C with 0.8 M HCl final concentration and neutralized through acid evaporation (N_2, 5h, 50 °C). Ultrapure water was added to the dry residue and two replicate samples were analyzed by Ion Chromatography (Dionex ICS 3000). The acidic sugars: galacturonic acid (2.8 µM, Gal-URA), gluconic acid (5.1 µM, Glu-Ac), glucuronic acid (3.0 µM, Glc-URA) and muramic acid (1.9 µM, Mur-Ac) were used as standards in a mixed solution. Regular calibration was performed by injecting 12.5 µl, 15.0 µl, 17.5 µl and 20 µl of mixed standard solution. Linearity of the calibration curves of individual sugar standards was verified in the concentration range 10 nM-10 µM. Therefore, the standard mixture was diluted 10, 20, 50, and 100 fold with Milli-Q water. Injection volume for samples and for the blank was 17.5 µl. The detection limit was 10 nM for each sugar with a precision of 13% estimated as the standard deviation of replicate measurements divided by the mean. Milli-Q water was used as blank to account for potential contamination during sample handling. Blanks were treated and analyzed in the same way as the samples. Blank concentration was subtracted from sample concentration if above the detection limit.

Marine gel particles: TEP and CSP

The size frequency distribution of marine gel particles was determined according to their ESD, described with a power function of the type:

$$dN/d(d_p) = k_p^{\delta}$$

where dN is the number of particles per unit volume in the size range d_p to $[d_p + d(d_p)]$, k is a constant which depends on the concentrations of particles, and δ is the spectral slope ($\delta < 0$) describing the size distribution. A less negative δ implies an increase in the fraction of larger marine gels. k and δ were both derived from regressions of $\log[dN/d(d_p)]$ versus $\log[d_p]$ [1-3]. The volume concentration of TEP and CSP refers to the mean volume of the particles > 0.4 µm which was the membrane pore size cutoff; changes in this parameter indicate particles dynamics such as aggregation/disaggregation processes. Since TEP are considered as fractal aggregates, the volume and the carbon content of these marine gel particles are assumed to be proportional to r^D, with r being the equivalent spherical radius (µm) and D the fractal scaling dimension associated with the size-distribution of marine gels [1,2,4]. TEP carbon content (TEP-C_{micro}, expressed in µM), was determined from marine gel size spectra according to Mari [5] and Engel [4]:

$$\text{TEP-}C_{micro}\ [\mu g\ L^{-1}] = 0.25 \times 10^{-6}\ r^D$$

with $D = 2.55$

References

1 Mari, X. & Burd, A. Seasonal size spectra of transparent exopolymeric particles (TEP) in a coastal sea and comparison with those predicted using coagulation theory. *Marine Ecology Progress Series* **163**, 63-76, doi:10.3354/meps163063 (1998).

2 Mari, X. & Kiørboe, T. Abundance, size distribution and bacterial colonization of transparent exopolymeric particles (TEP) during spring in the Kattegat. *Journal of Plankton Research* **18**, 969-986, doi:10.1093/plankt/18.6.969 (1996).

3 Harlay, J. *et al.* Abundance and size distribution of transparent exopolymer particles (TEP) in a coccolithophorid bloom in the northern Bay of Biscay. *Deep Sea Research Part I: Oceanographic Research Papers* **56**, 1251-1265, doi:10.1016/j.dsr.2009.01.014 (2009).

4 Engel, A. in *Practical Guidelines for the Analysis of Seawater* (ed. O. Wurl) Ch. 7, 125-142 (CRC Press, 2009).

5 Mari, X. Carbon content and C:N ratio of transparent exopolymeric particles (TEP) produced by bubbling exudates of diatoms. *Marine Ecology Progress Series* **183**, 59-71, doi:10.3354/meps183059(1999).

Supplementary Tables:

Table S1. Enrichment factors (EF, adimensional) and standard deviation (SD, %) per date, coordinates, depth, site and salinity for dissolved organic carbon (DOC), dissolved hydrolysable amino acids (DHAA), dissolved uronic acids (DURA), bacteria counts, and abundance of marine gels (as particles mL^{-1}) both as proteinaceous (CSP) and polysaccharidic particles (TEP). For TEP and CSP, we assumed a constant conservative SD of 50%.

Date	Lat. (N)	Long. (E)	Depth [cm]	Site	salinity [psu]	DOC	SD [%]	DHAA	SD [%]	DURA	SD [%]	Bacteria	SD [%]	CSP SD 50%	TEP SD 50%
10-Aug	84.05583	31.22361	30	t1	0.1	2.2	9	1.2	7	0.4	15			1.1	0.4
14-Aug	84.02056	76.11222	30	t1	n.a.	1.6	12	3.5	18	0.8	9			1.8	0.5
15-Aug	84.02056	76.11222	50	t1	n.a.	1.3	9	1.6	9	0.6	134	1.1	4.2	0.3	0.5
21-Aug	82.81333	109.6917	30	t1	0	1.1	4	1.4	8	0.5	19			1.5	0.6
10-Aug	84.05583	31.22361	open	t2	6.3	1.3	10	0.8	8	2.5	23	0.9	4.2	0.8	2.2
15-Aug	84.02056	76.11222	50	t2	n.a.	0.9	13	0.9	7	1.6	36	1.0	4.1	1.6	1.7
15-Aug	84.02056	76.11222	50	t2	n.a.	0.7	4	1.1	7	1.4	33	1.0	4.1	0.2	0.6
20-Aug	82.81333	109.6917	open	t2	n.a.	1.1	4	0.9	7	1.5	24			1.9	1.0
21-Aug	82.81333	109.6917	53	t2	0.6	1.1	3	1.2	7	0.9	17	0.7	4.6	0.7	1.6
21-Aug	82.81333	109.6917	40	t2	0	0.9	2	0.6	9	0.3	32	1.0	4.1	2.2	0.5
26-Aug	83.05139	129.9967	25	t2	1.7	1.6	2	2.8	14	0.4	53	0.8	4.4	2.0	0.7
26-Aug	83.05139	129.9967	20	t2	1.4	0.8	3	1.7	9	1.3	25	0.9	4.2	0.8	1.7
26-Aug	83.05139	129.9967	25	t2	28.8	0.5	2	1.6	9	0.4	35	0.5	6.3	3.1	1.3
05-Sep	82.09	131.1308	open	t3	30.02	1.4	1	4.1	20	1.9	17	0.7	4.7	4.0	1.2
05-Sep	82.09	131.1308	open	t3	30.2	1.0	2	1.7	9	0.9	9	1.2	4.3	2.8	1.5
07-Sep	85.23583	123.0417	open	t3	30	1.1	1	1.2	7	0.3	30	0.9	4.2	1.5	0.7
07-Sep	85.25139	122.9244	open	t3	29.9	0.7	2	1.9	10	0.8	25	0.9	4.2	2.7	1.4
07-Sep	85.27833	122.8014	open	t3	29.9	0.9	8	1.9	10	1.0	31	1.0	4.1	3.8	2.5
19-Sep	87.92972	61.12889	open	t3	n.a.	1.2	5	1.4	8	0.9	25	1.3	4.4	1.9	0.5
19-Sep	87.92972	61.12889	open	t3	n.a.	1.4	6	1.2	7	0.8	26	1.0	4.1	2.3	0.2
19-Sep	87.92972	61.12889	open	t3	30.6	1.9	9	1.4	8	1.4	31	1.0	4.1	3.8	0.5

Table S2. TEP and CSP particles abundance [10^3 particles mL^{-1}], contribution of small size fraction (0.4 – 1 μm) to total particles abundance, and volume concentration [ppm], in the SML and underlying water (ULW) of all sampling locations.

Site	CSP abundance [10^3 particles mL^{-1}]		CSP (0.4 – 1 μm) % of total abundance		CSP volume concentration [ppm]		TEP abundance [10^3 particles mL^{-1}]		TEP (0.4 – 1 μm) % of total abundance		TEP volume concentration [ppm]	
	**SML**	**ULW**	**SML**	**ULW**	**SML**	**ULW**	**SML**	**ULW**	**SML**	**ULW**	**SML**	**ULW**
**t1**	14	13	68.4	77.8	0.9	0.5	8	19	77.3	51.1	0.2	0.1
**t1**	29	17	73.8	81.9	0.6	0.2	7	14	74.1	69.6	0.2	0.2
**t1**	9	31	72.1	36.5	0.7	1.9	3	6	62.3	82.6	0.1	0.2
**t1**	32	21	61.4	62.4	1.7	1.6	13	23	82.3	65	0.3	0.4
**t2**	11	14	28.1	41.7	4.9	2.1	21	10	60.6	80.7	0.2	0.1
**t2**	40	26	46.3	58.2	1.4	0.9	13	7	79.1	83.7	0.6	0.2
**t2**	5	35	48.9	56.4	2	1.9	36	56	39.1	24.2	0.6	1.1
**t2**	26	13	59.9	58.2	3	7	28	26	47.8	59.1	0.4	0.9
**t2**	21	31	66.7	66.7	4.9	4.5	26	16	49.6	74	0.3	0.3
**t2**	22	10	71.4	73.1	1.9	2	23	47	55.4	33.4	0.7	0.4
**t2**	19	9	74	80.1	3	0.7	7	10	83.4	84.2	0.1	0.1
**t2**	8	9	77.4	78.8	0.4	0.7	14	8	83.6	84.3	0.1	0
**t2**	35	11	68.3	64.1	0.9	0.8	8	7	75	72.5	0.2	0.1
**t3**	33	8	44.2	52.2	2.3	0.9	30	24	45.1	40.2	0.5	0.3
**t3**	26	9	61.7	64.3	2.8	1.9	24	15	51.7	53.2	0.6	0.3
**t3**	20	13	65.1	70.6	2.7	2.8	13	18	64.9	77	0.3	0.4
**t3**	20	8	66.2	71.6	2.1	1.7	29	22	43.5	73.9	0.6	0.7
**t3**	53	14	53.9	64.4	9.1	2.8	48	20	24.5	71.8	1.1	0.7
**t3**	35	19	77	87	1.3	0.3	24	45	54.9	31.8	0.5	1.2
**t3**	34	15	77.4	77.5	1.7	1.6	6	35	75.3	39.5	0.9	0.2
**t3**	21	5	76.3	63.5	0.6	0.4	44	90	36.8	19.1	2.8	4.3

Table S3. Values for SML DOC concentration, dissolved hydrolysable amino acids (DHAA) and DHAA yields to DOC (DHAA-%DOC), dissolved uronic acids (DURA) and DURA yields to DOC (DURA-%DOC), and TEP carbon content as yield to TOC (TEP-%TOC).

**Date**	**Depth [cm]**	**Site**	**Ice thickness [cm]**	**salinity [psu]**	**DOC [µmol L^{-1}]**	**DHAA [nmol L^{-1}]**	**DHAA-%DOC**	**DURA [nmol L^{-1}]**	**DURA-%DOC**	**TEP-%TOC**
10-Aug	30	t1	120	0.1	10.3	101.3	3.8	33.0	1.9	1.1
14-Aug	30	t1	120	n.a.	18.5	440.1	9.1	19.2	0.6	1.3
15-Aug	50	t1	120	n.a.	15.9	215.7	5.1	69.4	2.6	0.6
21-Aug	30	t1	89	0	56.7	762.5	5.3	26.7	0.3	0.5
10-Aug	open	t2	120	6.3	16.0	294.3	7.5	16.4	0.6	1.0
15-Aug	50	t2	120	n.a.	13.2	149.6	4.3	18.5	0.8	2.3
15-Aug	50	t2	120	n.a.	15.5	211.1	5.2	53.6	2.1	2.2
20-Aug	open	t2	89	n.a.	52.7	455.0	3.3	76.5	0.9	0.7
21-Aug	53	t2	89	0.6	67.4	882.8	5.2	52.5	0.5	0.6
21-Aug	40	t2	89	0	58.6	533.0	3.7	8.2	0.1	0.7
26-Aug	25	t2	89	1.7	64.3	442.2	2.6	19.7	0.2	0.2
26-Aug	20	t2	89	1.4	45.3	268.0	2.4	26.2	0.3	0.3
26-Aug	25	t2	89	28.8	62.2	309.1	1.9	18.3	0.2	0.4
05-Sep	open	t3	89	30.02	169.8	838.8	1.8	51.0	0.2	0.4
05-Sep	open	t3	89	30.2	134.7	332.2	0.9	43.7	0.2	0.4
07-Sep	open	t3	81	30	145.7	262.7	0.6	25.5	0.1	0.3
07-Sep	open	t3	81	29.9	82.8	367.1	1.6	31.8	0.2	0.3
07-Sep	open	t3	81	29.9	91.3	365.9	1.5	43.1	0.3	0.8
19-Sep	open	t3	130	n.a.	104.6	308.9	1.1	38.7	0.2	0.6
19-Sep	open	t3	130	n.a.	110.2	164.1	0.5	23.3	0.1	0.3
19-Sep	open	t3	130	30.6	131.4	241.5	0.7	91.9	0.4	1.6

Supplementary Figures

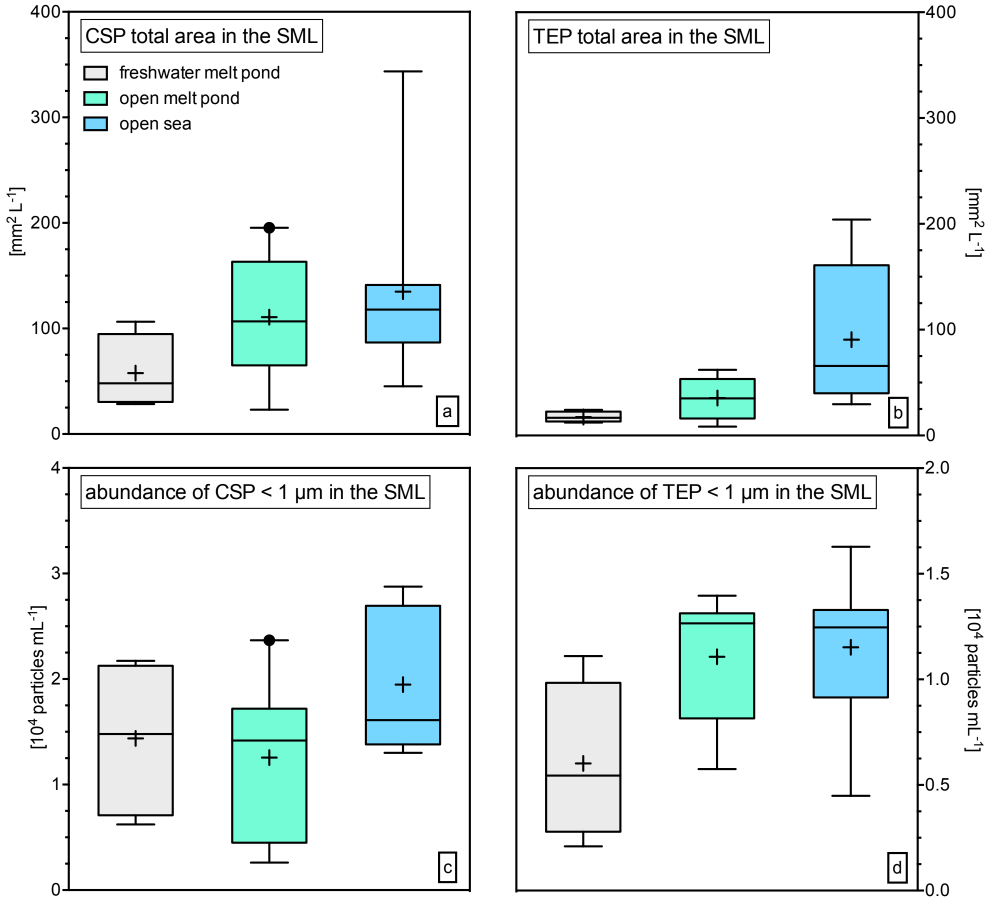

Figure S1a-d. Total area and abundance of gel particles in the SML. Total area [mm^2 L^{-1}]: (a) proteinaceous CSP, (b) polysaccharidic TEP. Abundance of submicron gel particles: (c) proteinaceous CSP, (d) polysaccharidic TEP. The horizontal lines of the boxes represent 25%, 50% (median) and 75% percentiles (from bottom to top). In the boxes, crosses represent the mean. Whiskers represent minimum and maximum values, and circles are outliers.

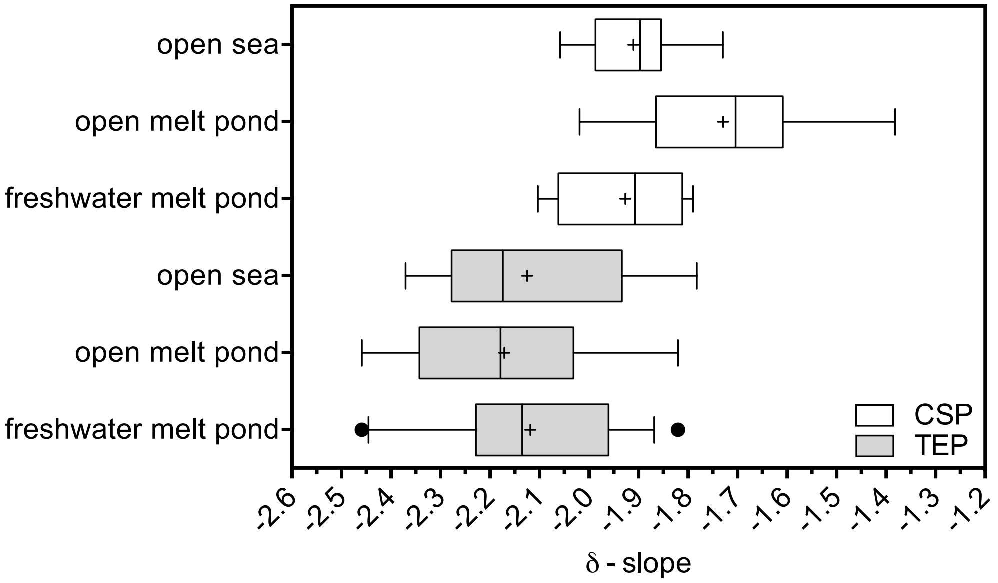

Figure S2. Slope (*δ*) of gel particles (TEP, CSP) in freshwater melt ponds, open melt ponds and open sea. A less negative slope implies a higher fraction of larger gel particles among the gel particles pool. The vertical lines of the boxes represent 25%, 50% (median) and 75% percentiles (from left to right). In the boxes, crosses represent the mean. Whiskers represent minimum and maximum values, and circles are outliers.
